# 2-Methyl-3-[(4-methyl­phen­yl)sulfon­yl­oxy]-2-{[(4-methyl­phen­yl)sulfon­yloxy]meth­yl}propyl 4-methyl­benzene­sulfonate

**DOI:** 10.1107/S1600536811024664

**Published:** 2011-06-30

**Authors:** Nassir N. Al-Mohammed, Raied M. Shakir, Yatimah Alias, Zanariah Abdullah, Siti Nadiah Abd Halim, Edward R. T. Tiekink

**Affiliations:** a Department of Chemistry, University of Malaya, 50603 Kuala Lumpur, Malaysia

## Abstract

The title mol­ecule, C_26_H_30_O_9_S_3_, adopts an extended conformation whereby two approximately parallel benzene rings [dihedral angle = 8.32 (10)°] are orientated in opposite directions along the pseudo-threefold axis through the central quaternary C atom, while a third ring occupies a position mid-way and face-on to these rings [dihedral angles = 82.28 (10) and 78.81 (7)°]. The crystal packing is dominated by C—H⋯O contacts and π–π inter­actions [ring centroid distance = 3.6902 (12) Å].

## Related literature

For the use of mol­ecules related to the title compound as synthetic precursors, see: Laliberte *et al.* (2003 ▶); Fujihara *et al.* (2007 ▶); Li *et al.* (2008*a* ▶,*b* ▶).
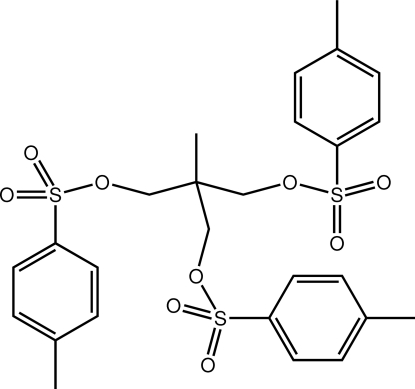


## Experimental

### 

#### Crystal data

C_26_H_30_O_9_S_3_
*M* *_r_* = 582.68
Triclinic, 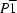

*a* = 10.2055 (3) Å
*b* = 12.4029 (3) Å
*c* = 12.7993 (4) Å
α = 66.868 (2)°
β = 78.370 (2)°
γ = 68.085 (2)°
*V* = 1379.32 (7) Å^3^
*Z* = 2
Mo *K*α radiation
μ = 0.32 mm^−1^
*T* = 100 K
0.30 × 0.28 × 0.28 mm

#### Data collection

Bruker SMART APEX diffractometer
Absorption correction: multi-scan (*SADABS*; Sheldrick, 1996 ▶) *T*_min_ = 0.636, *T*_max_ = 0.746
13040 measured reflections
6305 independent reflections
5369 reflections with *I* > 2σ(*I*)
*R*_int_ = 0.031

#### Refinement

*R*[*F* ^2^ > 2σ(*F* ^2^)] = 0.038
*wR*(*F* ^2^) = 0.117
*S* = 0.99
6303 reflections
347 parameters
H-atom parameters constrained
Δρ_max_ = 0.44 e Å^−3^
Δρ_min_ = −0.45 e Å^−3^

### 

Data collection: *APEX2* (Bruker, 2009 ▶); cell refinement: *SAINT* (Bruker, 2009 ▶); data reduction: *SAINT*; program(s) used to solve structure: *SHELXS97* (Sheldrick, 2008 ▶); program(s) used to refine structure: *SHELXL97* (Sheldrick, 2008 ▶); molecular graphics: *ORTEP-3* (Farrugia, 1997 ▶) and *DIAMOND* (Brandenburg, 2006 ▶); software used to prepare material for publication: *publCIF* (Westrip, 2010 ▶).

## Supplementary Material

Crystal structure: contains datablock(s) global, I. DOI: 10.1107/S1600536811024664/hg5057sup1.cif

Structure factors: contains datablock(s) I. DOI: 10.1107/S1600536811024664/hg5057Isup2.hkl

Supplementary material file. DOI: 10.1107/S1600536811024664/hg5057Isup3.cml

Additional supplementary materials:  crystallographic information; 3D view; checkCIF report

## Figures and Tables

**Table 1:** Hydrogen-bond geometry (Å, °)

*D*—H⋯*A*	*D*—H	H⋯*A*	*D*⋯*A*	*D*—H⋯*A*
C2—H2b⋯O2^i^	0.99	2.49	3.297 (2)	138
C4—H4a⋯O2^i^	0.99	2.42	3.290 (2)	147
C5—H5c⋯O8^ii^	0.98	2.54	3.440 (2)	152
C7—H7⋯O6^i^	0.95	2.54	3.183 (2)	125
C10—H10⋯O3^iii^	0.95	2.54	3.358 (2)	144
C15—H15⋯O9^iv^	0.95	2.56	3.428 (3)	151

Symmetry codes: (i) 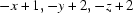; (ii) 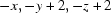; (iii) 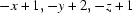; (iv) 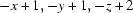.

**Fig. 1.**
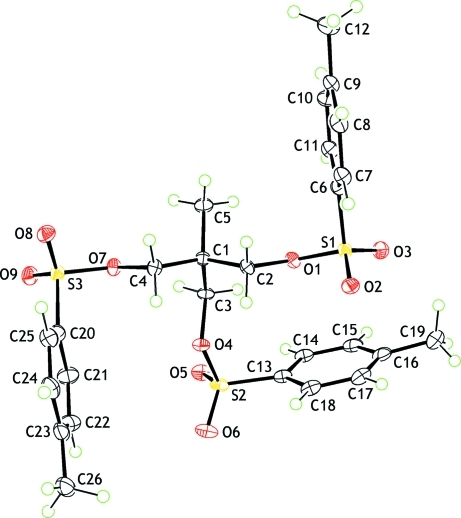
The molecular structure of compound (I) showing the atom-labelling scheme and displacement ellipsoids at the 50% probability level.

**Fig. 2.**
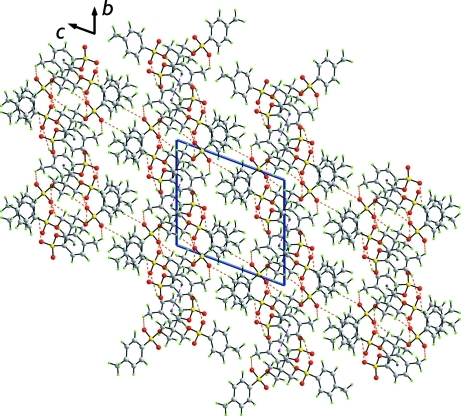
A view in projection down the a axis of the unit-cell contents for (I). The C—H···O and π–π interactions are shown as orange and purple dashed lines, respectively.

## supplementary crystallographic information

### Comment

Molecules related to the title compound, (I), are useful synthetic precursors for dendritic materials (Laliberte *et al.*, 2003), branched acyclic polyamines (Fujihara *et al.*, 2007) and radiopharmaceuticals (Li *et al.*, 2008*a*; Li *et al.*, 2008*b*).

With reference to the methyl group in the trisubstituted methane molecule, one benzene ring, connected to atom S1, is orientated in the same direction, and another, connected to S3, is approximately parallel but orientated in the opposite direction; dihedral angle = 8.32 (10) °. The third benzene ring lies approximately half-way between these rings and is face-on to each, forming dihedral angles of 82.28 (10) (S1) and 78.81 (7) °, respectively. This arrangement contrasts sharply the observed structure of the "parent" compound which adopts a somewhat flattened geometry with all benzene rings orientated in a circular manner around the central residue (Fujihara *et al.*, 2007).

The molecules are consolidated in the crystal structure by a combination of C—H···O, Table 1, and π–π interactions. The latter occur between centrosymmetrically related C13–C18 rings [3.6902 (12) Å for symmetry operation 2 - *x*, 1 - *y*, 2 - *z*]. Globally, layers of molecule interdigitate along the *c* axis, Fig. 2.

### Experimental

*p*-Toluenesulfonyl chloride (5.23 g, 27.4 mmol) in dry dichloromethane (50 ml) was added drop wise to a stirring solution of 1,1,1-tris(hydroxymethyl)ethane (1 g, 8.32 mmol) and triethylamine (5.05 g, 0.50 mmol) in dichloromethane (50 ml) at 273 K. The mixture was stirred at room temperature overnight, extracted with water, and wasted with distilled water (3 *x* 10 ml). The organic layer was dried over MgSO_4_ and evaporated. Colourless crystals were obtained from slow evaporation from its THF solution, *M*.pt 373–375 K.

### Refinement

Carbon-bound H-atoms were placed in calculated positions (C—H 0.93 to 0.99 Å) and were included in the refinement in the riding model approximation with *U_iso_*(H) = 1.2*U_eq_*(C) and 1.5*U_eq_*(methyl-C). Two reflections, *i.e*. (86 7) and (5 13 13), were omitted from the final refinement owing to poor agreement.

### Crystal data

**Table:**

C_26_H_30_O_9_S_3_	*Z* = 2
*M**_r_* = 582.68	*F*(000) = 612
Triclinic, *P*1	*D*_x_ = 1.403 Mg m^−^^3^
Hall symbol: -P 1	Mo *K*α radiation, λ = 0.71073 Å
*a* = 10.2055 (3) Å	Cell parameters from 5679 reflections
*b* = 12.4029 (3) Å	θ = 2.4–30.6°
*c* = 12.7993 (4) Å	µ = 0.32 mm^−^^1^
α = 66.868 (2)°	*T* = 100 K
β = 78.370 (2)°	Block, yellow
γ = 68.085 (2)°	0.30 × 0.28 × 0.28 mm
*V* = 1379.32 (7) Å^3^	

### Data collection

**Table:**

Bruker SMART APEX diffractometer	6305 independent reflections
Radiation source: fine-focus sealed tube	5369 reflections with *I* > 2σ(*I*)
graphite	*R*_int_ = 0.031
ω scans	θ_max_ = 27.5°, θ_min_ = 2.1°
Absorption correction: multi-scan (*SADABS*; Sheldrick, 1996)	*h* = −12→13
*T*_min_ = 0.636, *T*_max_ = 0.746	*k* = −16→16
13040 measured reflections	*l* = −16→16

### Refinement

**Table:**

Refinement on *F*^2^	Primary atom site location: structure-invariant direct methods
Least-squares matrix: full	Secondary atom site location: difference Fourier map
*R*[*F*^2^ > 2σ(*F*^2^)] = 0.038	Hydrogen site location: inferred from neighbouring sites
*wR*(*F*^2^) = 0.117	H-atom parameters constrained
*S* = 0.99	*w* = 1/[σ^2^(*F*_o_^2^) + (0.0689*P*)^2^ + 0.6349*P*] where *P* = (*F*_o_^2^ + 2*F*_c_^2^)/3
6303 reflections	(Δ/σ)_max_ = 0.001
347 parameters	Δρ_max_ = 0.44 e Å^−^^3^
0 restraints	Δρ_min_ = −0.45 e Å^−^^3^

### Special details

**Table:**

Geometry. All s.u.'s (except the s.u. in the dihedral angle between two l.s. planes) are estimated using the full covariance matrix. The cell s.u.'s are taken into account individually in the estimation of s.u.'s in distances, angles and torsion angles; correlations between s.u.'s in cell parameters are only used when they are defined by crystal symmetry. An approximate (isotropic) treatment of cell s.u.'s is used for estimating s.u.'s involving l.s. planes.
Refinement. Refinement of *F*^2^ against ALL reflections. The weighted *R*-factor *wR* and goodness of fit *S* are based on *F*^2^, conventional *R*-factors *R* are based on *F*, with *F* set to zero for negative *F*^2^. The threshold expression of *F*^2^ > 2σ(*F*^2^) is used only for calculating *R*-factors(gt) *etc*. and is not relevant to the choice of reflections for refinement. *R*-factors based on *F*^2^ are statistically about twice as large as those based on *F*, and *R*- factors based on ALL data will be even larger.

### Fractional atomic coordinates and isotropic or equivalent isotropic displacement parameters (Å^2^)

**Table:**

	*x*	*y*	*z*	*U*_iso_*/*U*_eq_	
S1	0.58792 (4)	0.97996 (4)	0.76513 (3)	0.01532 (11)	
S2	0.63629 (5)	0.55361 (4)	1.15173 (4)	0.01764 (12)	
S3	0.03815 (4)	0.75579 (4)	1.20789 (4)	0.01627 (11)	
O1	0.52171 (13)	0.87644 (11)	0.84519 (10)	0.0162 (3)	
O2	0.61934 (13)	1.03502 (12)	0.83218 (11)	0.0202 (3)	
O3	0.69823 (13)	0.91966 (12)	0.69899 (11)	0.0214 (3)	
O4	0.49589 (13)	0.66725 (11)	1.11596 (10)	0.0164 (3)	
O5	0.61861 (14)	0.44646 (11)	1.14753 (11)	0.0221 (3)	
O6	0.66462 (15)	0.55276 (12)	1.25683 (11)	0.0247 (3)	
O7	0.16219 (13)	0.74483 (11)	1.11229 (11)	0.0173 (3)	
O8	−0.07307 (13)	0.87019 (12)	1.16416 (11)	0.0217 (3)	
O9	0.01154 (14)	0.64130 (12)	1.24502 (11)	0.0221 (3)	
C1	0.33373 (18)	0.81398 (15)	0.97005 (14)	0.0148 (3)	
C2	0.41265 (18)	0.90728 (15)	0.93184 (14)	0.0156 (3)	
H2A	0.3469	0.9927	0.8998	0.019*	
H2B	0.4563	0.9016	0.9971	0.019*	
C3	0.43504 (18)	0.68148 (15)	1.01508 (14)	0.0152 (3)	
H3A	0.3835	0.6216	1.0353	0.018*	
H3B	0.5111	0.6653	0.9561	0.018*	
C4	0.22335 (18)	0.84493 (15)	1.06289 (15)	0.0166 (3)	
H4A	0.2681	0.8502	1.1218	0.020*	
H4B	0.1492	0.9254	1.0302	0.020*	
C5	0.26204 (19)	0.82440 (16)	0.87058 (15)	0.0190 (4)	
H5A	0.2030	0.7707	0.8988	0.029*	
H5B	0.3345	0.7986	0.8141	0.029*	
H5C	0.2028	0.9106	0.8352	0.029*	
C6	0.45022 (18)	1.08899 (16)	0.67862 (14)	0.0156 (3)	
C7	0.39715 (19)	1.21074 (16)	0.67582 (15)	0.0190 (4)	
H7	0.4322	1.2353	0.7232	0.023*	
C8	0.2920 (2)	1.29600 (17)	0.60257 (16)	0.0214 (4)	
H8	0.2547	1.3793	0.6004	0.026*	
C9	0.2403 (2)	1.26130 (17)	0.53222 (15)	0.0210 (4)	
C10	0.2951 (2)	1.13761 (17)	0.53788 (15)	0.0214 (4)	
H10	0.2598	1.1125	0.4911	0.026*	
C11	0.39924 (19)	1.05159 (17)	0.61026 (15)	0.0196 (4)	
H11	0.4357	0.9679	0.6134	0.023*	
C12	0.1290 (2)	1.3539 (2)	0.45091 (18)	0.0309 (5)	
H12A	0.1033	1.4355	0.4576	0.046*	
H12B	0.0451	1.3277	0.4692	0.046*	
H12C	0.1660	1.3590	0.3729	0.046*	
C13	0.75954 (18)	0.59940 (16)	1.04278 (15)	0.0182 (4)	
C14	0.81123 (19)	0.54084 (16)	0.96273 (16)	0.0201 (4)	
H14	0.7862	0.4714	0.9707	0.024*	
C15	0.89955 (19)	0.58496 (18)	0.87131 (17)	0.0229 (4)	
H15	0.9366	0.5443	0.8172	0.027*	
C16	0.93463 (19)	0.68791 (18)	0.85776 (17)	0.0237 (4)	
C17	0.8845 (2)	0.74337 (18)	0.94043 (18)	0.0246 (4)	
H17	0.9105	0.8122	0.9330	0.030*	
C18	0.79780 (19)	0.69997 (17)	1.03297 (17)	0.0214 (4)	
H18	0.7647	0.7381	1.0891	0.026*	
C19	1.0234 (2)	0.7391 (2)	0.75402 (19)	0.0309 (5)	
H19A	1.0906	0.6711	0.7302	0.046*	
H19B	1.0755	0.7811	0.7722	0.046*	
H19C	0.9620	0.7984	0.6921	0.046*	
C20	0.11684 (19)	0.76273 (16)	1.31382 (15)	0.0178 (3)	
C21	0.2287 (2)	0.66056 (18)	1.36842 (18)	0.0257 (4)	
H21	0.2603	0.5876	1.3496	0.031*	
C22	0.2925 (2)	0.66671 (19)	1.44988 (18)	0.0292 (4)	
H22	0.3678	0.5967	1.4880	0.035*	
C23	0.2490 (2)	0.77374 (19)	1.47790 (16)	0.0250 (4)	
C24	0.1370 (2)	0.87365 (18)	1.42254 (16)	0.0251 (4)	
H24	0.1051	0.9467	1.4412	0.030*	
C25	0.0706 (2)	0.86935 (17)	1.34066 (16)	0.0227 (4)	
H25	−0.0058	0.9387	1.3034	0.027*	
C26	0.3242 (2)	0.7802 (2)	1.56393 (17)	0.0331 (5)	
H26A	0.4148	0.7925	1.5292	0.050*	
H26B	0.3415	0.7027	1.6296	0.050*	
H26C	0.2655	0.8497	1.5890	0.050*	

### Atomic displacement parameters (Å^2^)

**Table:**

	*U*^11^	*U*^22^	*U*^33^	*U*^12^	*U*^13^	*U*^23^
S1	0.0169 (2)	0.0172 (2)	0.0134 (2)	−0.00673 (16)	−0.00021 (15)	−0.00623 (16)
S2	0.0226 (2)	0.0123 (2)	0.0173 (2)	−0.00169 (16)	−0.00767 (17)	−0.00503 (16)
S3	0.0153 (2)	0.0155 (2)	0.0178 (2)	−0.00525 (16)	−0.00088 (16)	−0.00542 (17)
O1	0.0194 (6)	0.0150 (6)	0.0148 (6)	−0.0057 (5)	0.0015 (5)	−0.0069 (5)
O2	0.0243 (7)	0.0240 (6)	0.0186 (6)	−0.0125 (5)	−0.0027 (5)	−0.0090 (5)
O3	0.0184 (6)	0.0251 (7)	0.0196 (6)	−0.0054 (5)	0.0018 (5)	−0.0098 (5)
O4	0.0190 (6)	0.0138 (6)	0.0157 (6)	−0.0016 (5)	−0.0058 (5)	−0.0058 (5)
O5	0.0288 (7)	0.0132 (6)	0.0244 (7)	−0.0042 (5)	−0.0077 (5)	−0.0061 (5)
O6	0.0349 (8)	0.0193 (6)	0.0192 (7)	−0.0033 (6)	−0.0123 (6)	−0.0060 (5)
O7	0.0179 (6)	0.0159 (6)	0.0202 (6)	−0.0077 (5)	0.0019 (5)	−0.0079 (5)
O8	0.0169 (6)	0.0211 (6)	0.0242 (7)	−0.0027 (5)	−0.0027 (5)	−0.0075 (5)
O9	0.0234 (7)	0.0213 (6)	0.0241 (7)	−0.0117 (5)	0.0004 (5)	−0.0074 (5)
C1	0.0162 (8)	0.0133 (7)	0.0151 (8)	−0.0041 (6)	−0.0025 (6)	−0.0051 (6)
C2	0.0184 (8)	0.0151 (8)	0.0130 (8)	−0.0051 (6)	0.0018 (6)	−0.0064 (6)
C3	0.0184 (8)	0.0138 (7)	0.0151 (8)	−0.0040 (6)	−0.0048 (6)	−0.0062 (6)
C4	0.0175 (8)	0.0131 (8)	0.0198 (8)	−0.0066 (6)	0.0008 (7)	−0.0059 (7)
C5	0.0207 (9)	0.0169 (8)	0.0203 (9)	−0.0030 (7)	−0.0063 (7)	−0.0079 (7)
C6	0.0178 (8)	0.0175 (8)	0.0115 (8)	−0.0066 (6)	0.0007 (6)	−0.0052 (6)
C7	0.0247 (9)	0.0187 (8)	0.0164 (8)	−0.0093 (7)	0.0011 (7)	−0.0080 (7)
C8	0.0257 (9)	0.0166 (8)	0.0199 (9)	−0.0063 (7)	0.0015 (7)	−0.0064 (7)
C9	0.0227 (9)	0.0218 (9)	0.0147 (8)	−0.0082 (7)	0.0000 (7)	−0.0024 (7)
C10	0.0244 (9)	0.0269 (9)	0.0162 (8)	−0.0097 (8)	−0.0025 (7)	−0.0091 (7)
C11	0.0233 (9)	0.0192 (8)	0.0177 (9)	−0.0071 (7)	−0.0003 (7)	−0.0085 (7)
C12	0.0318 (11)	0.0291 (10)	0.0245 (10)	−0.0062 (9)	−0.0080 (8)	−0.0027 (8)
C13	0.0177 (8)	0.0160 (8)	0.0208 (9)	−0.0013 (6)	−0.0073 (7)	−0.0075 (7)
C14	0.0192 (9)	0.0175 (8)	0.0238 (9)	−0.0007 (7)	−0.0091 (7)	−0.0086 (7)
C15	0.0184 (9)	0.0250 (9)	0.0245 (10)	0.0003 (7)	−0.0087 (7)	−0.0114 (8)
C16	0.0154 (8)	0.0261 (9)	0.0263 (10)	−0.0017 (7)	−0.0082 (7)	−0.0074 (8)
C17	0.0196 (9)	0.0226 (9)	0.0342 (11)	−0.0055 (7)	−0.0082 (8)	−0.0107 (8)
C18	0.0188 (9)	0.0200 (9)	0.0284 (10)	−0.0027 (7)	−0.0071 (7)	−0.0123 (8)
C19	0.0193 (10)	0.0401 (12)	0.0323 (11)	−0.0104 (9)	−0.0014 (8)	−0.0111 (10)
C20	0.0203 (8)	0.0187 (8)	0.0160 (8)	−0.0089 (7)	0.0004 (7)	−0.0061 (7)
C21	0.0262 (10)	0.0205 (9)	0.0292 (10)	−0.0035 (8)	−0.0086 (8)	−0.0083 (8)
C22	0.0301 (11)	0.0258 (10)	0.0287 (11)	−0.0073 (8)	−0.0119 (8)	−0.0035 (8)
C23	0.0305 (10)	0.0334 (10)	0.0164 (9)	−0.0207 (9)	0.0029 (8)	−0.0066 (8)
C24	0.0321 (10)	0.0264 (10)	0.0211 (9)	−0.0141 (8)	0.0050 (8)	−0.0118 (8)
C25	0.0253 (9)	0.0205 (9)	0.0214 (9)	−0.0074 (7)	0.0014 (7)	−0.0077 (7)
C26	0.0409 (12)	0.0497 (13)	0.0188 (10)	−0.0300 (11)	0.0008 (9)	−0.0092 (9)

### Geometric parameters (Å, °)

**Table:**

S1—O3	1.4241 (13)	C9—C12	1.502 (3)
S1—O2	1.4284 (13)	C10—C11	1.379 (3)
S1—O1	1.5780 (12)	C10—H10	0.9500
S1—C6	1.7498 (18)	C11—H11	0.9500
S2—O5	1.4286 (13)	C12—H12A	0.9800
S2—O6	1.4270 (13)	C12—H12B	0.9800
S2—O4	1.5809 (12)	C12—H12C	0.9800
S2—C13	1.7473 (19)	C13—C14	1.392 (2)
S3—O9	1.4219 (13)	C13—C18	1.395 (2)
S3—O8	1.4299 (13)	C14—C15	1.386 (3)
S3—O7	1.5799 (12)	C14—H14	0.9500
S3—C20	1.7530 (18)	C15—C16	1.389 (3)
O1—C2	1.4605 (19)	C15—H15	0.9500
O4—C3	1.4650 (19)	C16—C17	1.395 (3)
O7—C4	1.4606 (19)	C16—C19	1.507 (3)
C1—C2	1.523 (2)	C17—C18	1.381 (3)
C1—C4	1.525 (2)	C17—H17	0.9500
C1—C3	1.526 (2)	C18—H18	0.9500
C1—C5	1.533 (2)	C19—H19A	0.9800
C2—H2A	0.9900	C19—H19B	0.9800
C2—H2B	0.9900	C19—H19C	0.9800
C3—H3A	0.9900	C20—C25	1.384 (2)
C3—H3B	0.9900	C20—C21	1.394 (3)
C4—H4A	0.9900	C21—C22	1.375 (3)
C4—H4B	0.9900	C21—H21	0.9500
C5—H5A	0.9800	C22—C23	1.399 (3)
C5—H5B	0.9800	C22—H22	0.9500
C5—H5C	0.9800	C23—C24	1.387 (3)
C6—C11	1.391 (2)	C23—C26	1.505 (3)
C6—C7	1.389 (2)	C24—C25	1.385 (3)
C7—C8	1.389 (3)	C24—H24	0.9500
C7—H7	0.9500	C25—H25	0.9500
C8—C9	1.393 (3)	C26—H26A	0.9800
C8—H8	0.9500	C26—H26B	0.9800
C9—C10	1.400 (3)	C26—H26C	0.9800
			
O3—S1—O2	120.03 (8)	C10—C9—C12	120.15 (18)
O3—S1—O1	104.03 (7)	C11—C10—C9	121.12 (17)
O2—S1—O1	109.40 (7)	C11—C10—H10	119.4
O3—S1—C6	109.49 (8)	C9—C10—H10	119.4
O2—S1—C6	109.38 (8)	C10—C11—C6	119.11 (16)
O1—S1—C6	103.10 (7)	C10—C11—H11	120.4
O5—S2—O6	119.61 (8)	C6—C11—H11	120.4
O5—S2—O4	108.93 (7)	C9—C12—H12A	109.5
O6—S2—O4	104.27 (7)	C9—C12—H12B	109.5
O5—S2—C13	109.07 (8)	H12A—C12—H12B	109.5
O6—S2—C13	111.07 (9)	C9—C12—H12C	109.5
O4—S2—C13	102.39 (7)	H12A—C12—H12C	109.5
O9—S3—O8	119.98 (8)	H12B—C12—H12C	109.5
O9—S3—O7	104.01 (7)	C14—C13—C18	120.74 (18)
O8—S3—O7	108.91 (7)	C14—C13—S2	119.90 (14)
O9—S3—C20	110.37 (8)	C18—C13—S2	119.23 (14)
O8—S3—C20	109.18 (8)	C13—C14—C15	119.28 (17)
O7—S3—C20	102.94 (8)	C13—C14—H14	120.4
C2—O1—S1	117.04 (10)	C15—C14—H14	120.4
C3—O4—S2	115.37 (10)	C14—C15—C16	120.83 (18)
C4—O7—S3	117.67 (10)	C14—C15—H15	119.6
C2—C1—C4	106.13 (13)	C16—C15—H15	119.6
C2—C1—C3	110.89 (14)	C15—C16—C17	118.95 (19)
C4—C1—C3	111.03 (14)	C15—C16—C19	119.98 (18)
C2—C1—C5	110.90 (14)	C17—C16—C19	121.06 (19)
C4—C1—C5	110.44 (14)	C18—C17—C16	121.14 (18)
C3—C1—C5	107.49 (13)	C18—C17—H17	119.4
O1—C2—C1	106.76 (13)	C16—C17—H17	119.4
O1—C2—H2A	110.4	C17—C18—C13	118.99 (17)
C1—C2—H2A	110.4	C17—C18—H18	120.5
O1—C2—H2B	110.4	C13—C18—H18	120.5
C1—C2—H2B	110.4	C16—C19—H19A	109.5
H2A—C2—H2B	108.6	C16—C19—H19B	109.5
O4—C3—C1	108.11 (12)	H19A—C19—H19B	109.5
O4—C3—H3A	110.1	C16—C19—H19C	109.5
C1—C3—H3A	110.1	H19A—C19—H19C	109.5
O4—C3—H3B	110.1	H19B—C19—H19C	109.5
C1—C3—H3B	110.1	C25—C20—C21	120.65 (18)
H3A—C3—H3B	108.4	C25—C20—S3	120.30 (15)
O7—C4—C1	106.51 (13)	C21—C20—S3	119.03 (14)
O7—C4—H4A	110.4	C22—C21—C20	119.15 (18)
C1—C4—H4A	110.4	C22—C21—H21	120.4
O7—C4—H4B	110.4	C20—C21—H21	120.4
C1—C4—H4B	110.4	C21—C22—C23	121.48 (19)
H4A—C4—H4B	108.6	C21—C22—H22	119.3
C1—C5—H5A	109.5	C23—C22—H22	119.3
C1—C5—H5B	109.5	C24—C23—C22	118.02 (18)
H5A—C5—H5B	109.5	C24—C23—C26	121.39 (19)
C1—C5—H5C	109.5	C22—C23—C26	120.57 (19)
H5A—C5—H5C	109.5	C25—C24—C23	121.51 (17)
H5B—C5—H5C	109.5	C25—C24—H24	119.2
C11—C6—C7	121.17 (17)	C23—C24—H24	119.2
C11—C6—S1	118.32 (13)	C24—C25—C20	119.18 (18)
C7—C6—S1	120.47 (14)	C24—C25—H25	120.4
C6—C7—C8	118.89 (17)	C20—C25—H25	120.4
C6—C7—H7	120.6	C23—C26—H26A	109.5
C8—C7—H7	120.6	C23—C26—H26B	109.5
C9—C8—C7	121.08 (16)	H26A—C26—H26B	109.5
C9—C8—H8	119.5	C23—C26—H26C	109.5
C7—C8—H8	119.5	H26A—C26—H26C	109.5
C8—C9—C10	118.62 (17)	H26B—C26—H26C	109.5
C8—C9—C12	121.23 (17)		
			
O3—S1—O1—C2	174.43 (11)	C7—C6—C11—C10	0.6 (3)
O2—S1—O1—C2	45.02 (13)	S1—C6—C11—C10	−177.19 (13)
C6—S1—O1—C2	−71.29 (13)	O5—S2—C13—C14	6.56 (17)
O5—S2—O4—C3	−45.30 (13)	O6—S2—C13—C14	140.46 (14)
O6—S2—O4—C3	−174.07 (11)	O4—S2—C13—C14	−108.75 (15)
C13—S2—O4—C3	70.11 (12)	O5—S2—C13—C18	−177.62 (14)
O9—S3—O7—C4	−174.81 (12)	O6—S2—C13—C18	−43.72 (16)
O8—S3—O7—C4	56.15 (13)	O4—S2—C13—C18	67.08 (15)
C20—S3—O7—C4	−59.63 (13)	C18—C13—C14—C15	−1.2 (3)
S1—O1—C2—C1	161.94 (11)	S2—C13—C14—C15	174.54 (13)
C4—C1—C2—O1	178.34 (13)	C13—C14—C15—C16	−1.2 (3)
C3—C1—C2—O1	57.66 (17)	C14—C15—C16—C17	2.8 (3)
C5—C1—C2—O1	−61.69 (17)	C14—C15—C16—C19	−176.23 (17)
S2—O4—C3—C1	−161.46 (11)	C15—C16—C17—C18	−2.0 (3)
C2—C1—C3—O4	63.10 (17)	C19—C16—C17—C18	177.03 (17)
C4—C1—C3—O4	−54.63 (18)	C16—C17—C18—C13	−0.4 (3)
C5—C1—C3—O4	−175.53 (13)	C14—C13—C18—C17	2.0 (3)
S3—O7—C4—C1	−177.15 (11)	S2—C13—C18—C17	−173.78 (14)
C2—C1—C4—O7	−171.40 (13)	O9—S3—C20—C25	−135.36 (15)
C3—C1—C4—O7	−50.81 (18)	O8—S3—C20—C25	−1.46 (18)
C5—C1—C4—O7	68.33 (17)	O7—S3—C20—C25	114.13 (15)
O3—S1—C6—C11	49.51 (16)	O9—S3—C20—C21	46.18 (17)
O2—S1—C6—C11	−177.08 (13)	O8—S3—C20—C21	−179.91 (14)
O1—S1—C6—C11	−60.76 (15)	O7—S3—C20—C21	−64.32 (16)
O3—S1—C6—C7	−128.28 (14)	C25—C20—C21—C22	0.2 (3)
O2—S1—C6—C7	5.13 (17)	S3—C20—C21—C22	178.66 (16)
O1—S1—C6—C7	121.45 (14)	C20—C21—C22—C23	−0.9 (3)
C11—C6—C7—C8	−0.4 (3)	C21—C22—C23—C24	1.2 (3)
S1—C6—C7—C8	177.30 (13)	C21—C22—C23—C26	−177.53 (19)
C6—C7—C8—C9	−0.4 (3)	C22—C23—C24—C25	−0.9 (3)
C7—C8—C9—C10	1.0 (3)	C26—C23—C24—C25	177.89 (18)
C7—C8—C9—C12	−178.54 (17)	C23—C24—C25—C20	0.2 (3)
C8—C9—C10—C11	−0.8 (3)	C21—C20—C25—C24	0.1 (3)
C12—C9—C10—C11	178.70 (17)	S3—C20—C25—C24	−178.28 (14)
C9—C10—C11—C6	0.1 (3)		

### Hydrogen-bond geometry (Å, °)

**Table:**

*D*—H···*A*	*D*—H	H···*A*	*D*···*A*	*D*—H···*A*
C2—H2b···O2^i^	0.99	2.49	3.297 (2)	138
C4—H4a···O2^i^	0.99	2.42	3.290 (2)	147
C5—H5c···O8^ii^	0.98	2.54	3.440 (2)	152
C7—H7···O6^i^	0.95	2.54	3.183 (2)	125
C10—H10···O3^iii^	0.95	2.54	3.358 (2)	144
C15—H15···O9^iv^	0.95	2.56	3.428 (3)	151

Symmetry codes: (i) −*x*+1, −*y*+2, −*z*+2; (ii) −*x*, −*y*+2, −*z*+2; (iii) −*x*+1, −*y*+2, −*z*+1; (iv) −*x*+1, −*y*+1, −*z*+2.
